# Wideband Multichannel Nyquist-Spaced Long-Haul Optical Transmission Influenced by Enhanced Equalization Phase Noise

**DOI:** 10.3390/s23031493

**Published:** 2023-01-29

**Authors:** Cenqin Jin, Nikita A. Shevchenko, Junqiu Wang, Yunfei Chen, Tianhua Xu

**Affiliations:** 1 School of Engineering, University of Warwick, Coventry CV4 7AL, UK; 2 Department of Electronic & Electrical Engineering, University College London (UCL), London WC1E 7JE, UK; 3 Department of Engineering, Durham University, Durham DH1 3LE, UK

**Keywords:** optical fiber communication, equalization enhanced phase noise, digital nonlinearity compensation, Gaussian noise model, laser phase noise, electronic dispersion compensation

## Abstract

Enhanced equalization phase noise (EEPN), generated from the uncompensated dispersion experienced by laser phase noises, can cause serious damage to the transmission quality of optical fiber systems. In this work, the performance of a wideband Nyquist-spaced long-haul nonlinear optical fiber communication systems suffering from EEPN is investigated and discussed through split-step numerical simulations and analytical models based on the perturbation analysis, in the cases of digital nonlinearity compensation (NLC) and electronic dispersion compensation (EDC). The efficiency and the accuracy of the analytical models were validated via simulations, considering the different symbol rates and modulation formats. The performance of the *C*-band transmission was comprehensively studied based on the model. Our results reveal that the growth of symbol rates and transmission distances aggravates the distortions in the *C*-band system.

## 1. Introduction

With the development of modern society and the mobile Internet, especially after the COVID-19 outbreak, the demand for the communication capacity has increased significantly, leading to higher requirements for the transmission quality of long-distance and high-speed communications. Nyquist-spaced optical fiber transmission is used to improve the spectral efficiency, although it is significantly affected by various distortions, including polarization mode dispersion (PMD), chromatic dispersion (CD), fiber nonlinearity from the Kerr effect, laser phase noise (LPN) and other effects [1]. These can be compensated by digital signal processing (DSP) approaches [2]. Nevertheless, distortions generated by interactions between LPNs and fiber dispersion, i.e., enhanced equalization phase noises (EEPNs), are difficult to effectively compensate at present [1,3]. The EEPN effects have attracted increasing research attention from academic and industrial institutions in recent years [4,5,6,7,8,9]. The perturbation-based Guassian noise (GN) model is a convenient and sufficiently accurate method to predict the quality of signal transmission over long-haul fiber systems [10,11,12]. Traditional GN models do not consider the influence of EEPN. In our previous work, we improved the traditional GN models, taking the EEPN effects into consideration, for 32-GHz Nyquist-spaced nonlinear optical fibre systems [13]. However, to the best of our knowledge, the EEPN effects in wideband transmission systems have not been investigated to date, and the impact of symbol rate on the performance of transmissions influenced by EEPN has not been analysed and discussed. The EEPN scales according to increments in LPN, transmission distance, symbol rate, etc. [14,15], and can seriously degrade the system transmission performance [13,16,17,18]. Therefore, it is necessary to study the long-distance, wideband, high-speed Nyquist communication system considering the influence of EEPN.

Compared to our previous work [13], which provided only a simplified closed-form expression, we present detailed and generic formulas to calculate the nonlinear coefficients in integral form in this paper, with improved accuracy. The presentation of analytical models is more systematic and comprehensive. The impact of the transmission symbol rate is also taken into account. The symbol rates of 16, 32, 64 and 128 GBd are considered, while only the 32 GBd optical system was considered in [13]. The results reveal that the growth of symbol rates aggravates the distortions. Meanwhile, the performance of the *C*-band transmission is studied, based on the analytical estimation. The considered bandwidth was extended to 4.5 THz to evaluate the performance of an entire *C*-band transmission system.

Overall, in this paper, we investigated the performance of long-haul Nyquist-spaced multichannel optical communication systems, based on numerical simulations and the GN model and considering the EEPN effects in scenarios of nonlinearity compensation (NLC) and electronic dispersion compensation (EDC). The performance of the *C*-band transmission considering the significant LPN was assessed and analyzed based on the analytical model. The factors of transmission symbol rate, modulation format, and transmission distance were comprehensively taken into account.

The paper has been organized as follows: Section 2 explains the origin of EEPN effects and the theoretical analyses. Section 3 presents the analytical GN model approach to evaluating the Nyquist-spaced nonlinear fiber transmission influenced by EEPN effects. Section 4 describes the transmission system setup. Section 5 presents and discusses simulated and analytical results. Section 6 is the conclusion.

## 2. Enhanced Equalization Phase Noise

**Figure 1:**
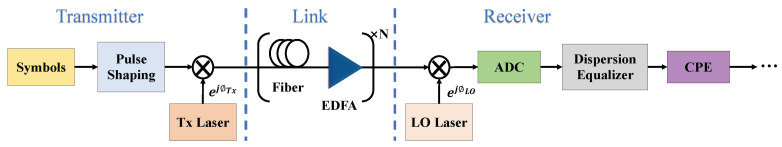
Principle of EEPN in optical fiber transmission.

EEPN effects are generated by the interaction between CDs and LPNs from a local oscillator (LO) or a transmitter (Tx) laser source [17,19]. A general dispersion-unmanaged nonlinear optical fiber coherent system, which contains Tx, a transmission link, and a coherent receiver (Rx), is shown in Figure 1. At the Tx, the symbol sequence experiences a pulse-shaping filter to create a band-limited continuous signal sequence. When modulated on the Tx laser carrier, the Tx LPN is introduced to the signal. This LPN is first dispersed in the fiber link, and is then compensated by the dispersion equalizer at the receiver. However, the LO laser also produces the LPN, which only interacts with the electronic dispersion equalizer. The net dispersion arising from the LPN of LO laser in this scenario generates the EEPN [16]. Similarly, the EEPN can be caused by the interference between the Tx LPN and the fiber dispersion [19,20] when the received signals pass through a carrier phase estimation (CPE), firstly for the LPN mitigation, and then experience the dispersion equalizer. Since the DSP scheme where EEPN originates from the LO LPN is more common, this scenario is discussed in this paper.

The effect of EEPN is related to the transmission parameters, including the accumulated CD, the transmission bandwidth, and the laser linewidth. The variance in EEPN can be expressed as [17,21]

(1) $$\sigma_{\mathrm{EEPN}}^{2}\left(L\right)=N\;\frac{\pi c\;D\;L\;f_{3\mathrm{dB}}}{2f_{0}^{2}}\cdot R\;,$$

where *D* represents the chromatic dispersion coefficient, *N* denotes the span number, *c* denotes the light speed in vacuum, $f_{0}$ means the laser center frequencies, *L* is the fiber span length, $f_{3\mathrm{dB}}$ is 3-dB laser linewidths, and *R* denotes the transmitted symbol rate.

## 3. Analytical Model

The analytical model for the performance prediction of multichannel long-haul Nyquist-spaced fiber communications, influenced by EEPN in terms of signal-to-noise ratio (SNR), is presented in this section.

The SNR of dispersion-unmanaged systems influenced by EEPN is given by the following expression, based on the GN model [22,23]

(2) $$\mathrm{SNR}=\frac{P}{P_{\mathrm{ASE}}+P_{s-s}+P_{s-\mathrm{ASE}}+P_{\mathrm{EEPN}}+P_{s-\mathrm{EEPN}}}\;,$$

where *P* stands for the launch power per channel, $P_{s-s}$ represents the signal–signal nonlinear interaction, $P_{\mathrm{ASE}}$ means amplified spontaneous emission (ASE) noises from erbium-doped fiber amplifiers (EDFAs), $P_{s-\mathrm{ASE}}$ denotes the signal–ASE interference arising from four-wave mixing processes, $P_{\mathrm{EEPN}}$ evaluates the EEPN effect, and $P_{s-\mathrm{EEPN}}$ is the nonlinear interference between EEPN and signal. For a dual-polarization nonlinear long-haul optical transmission, these are given by the following expressions:

(3) $$P_{\mathrm{ASE}}=N\left(G-1\right)F_{n}\;hf_{0}\cdot R\;,$$

(4) $$P_{s-s}=\eta \left(N,B\right)\cdot P^{3}\;,$$

(5) $$P_{s-\mathrm{ASE}}\approx 3\;\xi_{1}\;\eta \left(1,B\right)P_{\mathrm{ASE}}\cdot P^{2}+9\;\xi_{2}\;\eta {\left(1,B\right)}^{2}P_{\mathrm{ASE}}\cdot P^{4}\;,$$

(6) $$P_{\mathrm{EEPN}}=\sigma_{\mathrm{EEPN}}^{2}\cdot P\;,$$

(7) $$P_{s-\mathrm{EEPN}}=3\;\xi_{1}\;\eta \left(1,B\right)\;\left(\sigma_{\mathrm{EEPN}}^{2}/N\right)\cdot P^{3},$$

where *G* denotes the EDFA gain, *h* is the Planck constant, $F_{n}$ stands for the noise figure of EDFA, $\eta \left(N,B\right)$ denotes the nonlinear interference (NLI) distortion coefficient with the transmitted bandwidth *B*, $\eta \left(1,B\right)$ is the NLI distortion coefficient of single span, $\xi_{1}≜\sum_{n=1}^{N}n^{\varepsilon +1}$ (see, e.g., [10,11,24]), and $\xi_{2}≜\sum_{n=2}^{N}\;\sum_{m=1}^{n-1}\;m^{\varepsilon +1}$ (see [25]). with ε being the coherence factor [23].

Assuming that all wavelength division multiplexing (WDM) channels possess the same dual-polarization multiplexed modulation format, the NLI coefficient $\eta \left(N,B\right)$ evaluated at the center channel can be decomposed as follows

(8) $$\eta \left(N,B\right)=\eta^{\left(0\right)}\left(N,B\right)+\eta^{\left(\mathrm{QAM}\right)}\left(N,B\right).$$

The first term $\eta^{\left(0\right)}\left(N,B\right)$ in Equation (8) is the signal modulation format independent term, which evaluates the NLI noise contribution assuming a Gaussian input. In Nyquist-spaced WDM systems, this is given by the following double integral [10,23,26,27]

(9) $$\eta^{\left(0\right)}\left(N,B\right)=\frac{16\gamma^{2}}{27\;R^{2}}\;\int_{-B/2}^{B/2}\;\int_{-B/2}^{B/2}\;df_{1}df_{2}\;{\left|\;\varphi \left(f,f_{1},f_{2}\;|\;L,N\right)\cdot \rho \left(f,f_{1},f_{2}\;|\;L\right)\right|}^{\;2}\;\mathrm{rect}\left(\frac{f_{1}+f_{2}}{B}\right),$$

where γ is the fiber nonlinear coefficient, $\mathrm{rect}\left(x\right)$ stands for the rectangular function, the factor $\varphi \left(f,f_{1},f_{2}\;|\;N\right)$ accounts for the NLI distance evolution over multi-span fiber transmission, and $\rho \left(f,f_{1},f_{2}\;|\;L\right)$ is the four-wave mixing efficiency factor. These factors have the following closed-form expressions:

(10) $$\varphi \left(f,f_{1},f_{2}\;|\;L,N\right)=\frac{1-\exp\left(\;i\;\Delta \beta \left(f,f_{1},f_{2}\right)\cdot NL\right)}{1-\exp\left(\;i\;\Delta \beta \left(f,f_{1},f_{2}\right)\cdot L\right)},$$

(11) $$\rho \left(f,f_{1},f_{2}\;|\;L\right)=\;\frac{1-\exp\left[-\left(\alpha +i\;\Delta \beta \left(f,f_{1},f_{2}\right)\right)\cdot L\;\right]}{\alpha -i\;\Delta \beta \left(f,f_{1},f_{2}\right)},$$

where $i≜\sqrt{-1}$ denotes the imaginary unit, and the four-wave mixing phase-mismatch $\Delta \beta \left(f,f_{1},f_{2}\right)$ can be approximated as (see, e.g., [28])

(12) $$\begin{array}{c} \Delta \beta \left(f,f_{1},f_{2}\right)\approx 4\pi^{2}\left[\;\beta_{2}+\pi \left(f_{1}+f_{2}\right)\beta_{3}\;\right]\cdot \left(f_{1}-f\right)\left(f_{2}-f\right), \end{array}$$

where $\beta_{2}$ and $\beta_{3}$ are the 2nd- and the 3rd-order dispersion coefficients, respectively [10,29].

The second term $\eta^{\left(\mathrm{QAM}\right)}\left(N,B\right)$ in Equation (8) includes the corrections needed for the input QAM format. It is customary to use the following closed-form approximation [30]

(13) $$\eta^{\left(\mathrm{QAM}\right)}\left(N,B\right)\approx -\frac{80}{81}\;\chi \;\frac{N\gamma^{2}L_{\mathrm{eff}}^{2}}{\pi \left|\beta_{2}\right|L\;R^{2}}\left[\mathrm{HN}\left(\frac{B/R-1}{2}\right)+1\right]\cdot P^{3}\;,$$

where $L_{\mathrm{eff}}$ stands for the effective length of fiber span, χ denotes the constant pre-factor, and is related to the excess kurtosis of input QAM signal modulation format. The values of χ for the quadrature phase shift keying (QPSK), 16QAM, 32QAM, 64QAM, and Gaussian input are equal to {1,17/25,69/100,13/21,0}, respectively. Finally, the function $\mathrm{HN}\left(x\right)$ denotes harmonic numbers, and can be expressed by $\sum_{n=1}^{x}1/n$.

When only EDC is employed in systems, the contribution of $P_{s-\mathrm{ASE}}$ is negligible compared with the $P_{s-s}$. In systems with NLC where $P_{s-s}$ is considerably reduced, $P_{s-\mathrm{ASE}}$ becomes comparatively significant. When the full-field NLC (which is employed in all signal bandwidths) is considered in this paper, $P_{s-s}$ can be fully removed. Therefore, the model SNR expressions in the presence of EEPN in scenarios of EDC and NLC are, respectively, given by

(14) $${\mathrm{SNR}}_{\mathrm{EDC}}=\frac{P}{P_{\mathrm{ASE}}+P_{s-s}+P_{\mathrm{EEPN}}}\;,$$

(15) $${\mathrm{SNR}}_{\mathrm{NLC}}=\frac{P}{P_{\mathrm{ASE}}+P_{s-\mathrm{ASE}}+P_{\mathrm{EEPN}}+P_{s-\mathrm{EEPN}}}\;.$$

Based on Equations (14) and (15), when EDC and NLC employed, respectively, their optimal launch powers, corresponding maximum SNR values can be estimated by

(16) $$P_{\mathrm{EDC},\mathrm{opt}}=\sqrt[{3}]{\frac{P_{\mathrm{ASE}}}{2N^{\varepsilon }\;\eta \left(1,B\right)}}\;,$$

(17) $$\max_{P}\;\left[\;{\mathrm{SNR}}_{\mathrm{EDC}}\;\right]=\frac{1}{\sigma_{\mathrm{EEPN}}^{2}+\sqrt[{3}]{\frac{27}{4}N^{\varepsilon +3}\;\eta \left(1,B\right)\;P_{\mathrm{ASE}}^{2}}}\;,$$

(18) $$P_{\mathrm{NLC},\mathrm{opt}}\approx \sqrt{\frac{N\;}{3\xi_{1}\;\eta \left(1,B\right)}}\;,$$

(19) $$\max_{P}\;\left[\;{\mathrm{SNR}}_{\mathrm{NLC}}\;\right]\approx \frac{1}{\sigma_{\mathrm{EEPN}}^{2}+\sqrt{12\xi_{1}N\eta \left(1,B\right)P_{\mathrm{ASE}}^{2}}}\;.$$

## 4. Transmission System

**Figure 2:**
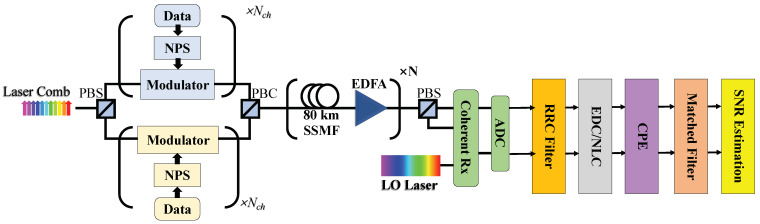
Scheme of the dual-polarization Nyquist-spaced multichannel communication system. PBS: polarization beam-splitter; PBC: polarization beam combiner; NPS: Nyquist pulse-shaping.

The performances of long-distance Nyquist-spaced multichannel nonlinear systems has been investigated by numerical simulations. Figure 2 shows the simulated system scheme. At the Tx, optical carriers are generated by a laser comb. The signal symbol sequences in different channels were generated independently and randomly, and are shaped by root-raised cosine (RRC) filters. In the transmission link, standard single mode fibers (SSMFs) were applied, where each fiber spans 80 km. EDFAs with the noise figure of 4.5 dB were applied to completely compensate the loss after fiber spans. Split-step Fourier method for solving Manakov equations was employed for fiber signal transmission [31,32]. After the fiber transmission link, coherent detection was implemented by a 100 kHz linewidth LO laser. In DSP modules, a roll-off RRC filter was employed to select NLC bandwidths. The EDC was realized by a equalization in frequency domain [29], and the NLC was implemented via the inverse split-step Fourier simulation [33]. To focus on the EEPN influence, the LPN from LO was recorded and compensated by an ideal CPE [19]. Before the assessment of system performance in terms of SNR, the observed channel was selected by the matched filter. The influence of laser frequency offset and PMD was ignored. Main simulation parameters are listed in Table 1.

## 5. Results and Discussions

This section describes the analytical and simulated results for WDM Nyquist-spaced nonlinear coherent optical fiber systems. The prediction for *C*-band systems wa salso made, based on the analytical model. The impact of different modulation formats, as well as the transmission rates and distances, are considered and discussed.

**Figure 3:**
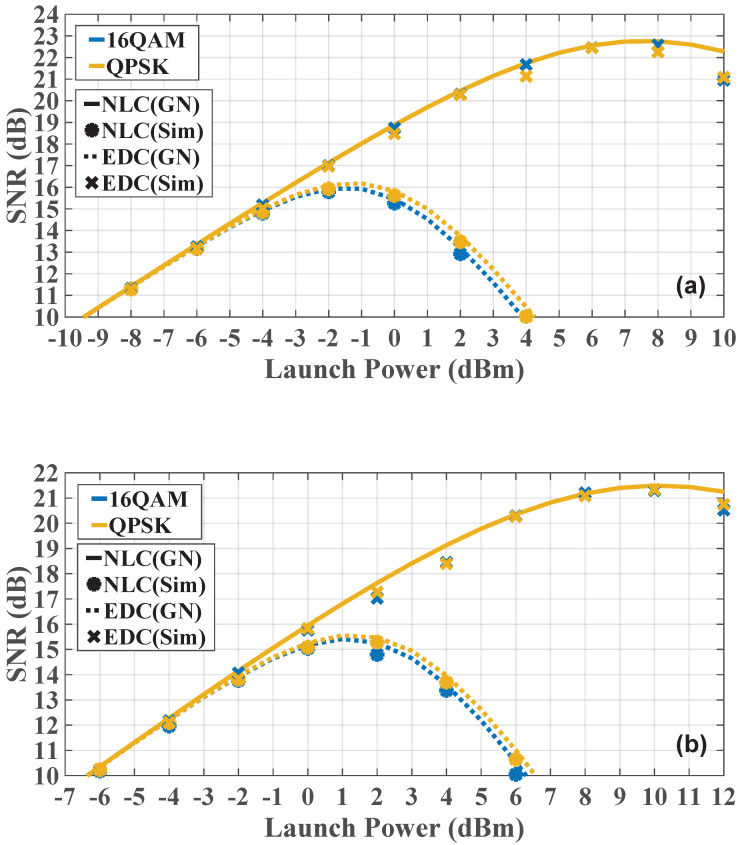
The SNR with different launch power in Nyquist-spaced 5-channel systems with transmission symbol rates of 32 GBd in (**a**), and of 64 GBd in (**b**).

Figure 3 shows the central channel SNR with varying launch powers in a Nyquist-spaced 25×80 km 5-channel system with 32-GBd transmission symbol rate in (a), with 64-GBd transmission rate in (b), where significant LO LPN with 100 kHz linewidth is considered. The dotted line represents the result of EDC model Equation (14), and the solid line represents the results of NLC model Equation (15). Both DP-QPSK and DP-16QAM modulation formats are considered. The great consistency between the results of analytical model and simulation is shown in Figure 3a,b, which validates the efficiency and the accuracy of the model described in Section 3 in 32-GBd and 64-GBd DP-QPSK and DP-16QAM multichannel Nyquist-spaced nonlinear fiber transmission in cases of NLC and EDC. It is also be observed that the QPSK systems can achieve a better performance than 16QAM in the case of EDC. This is because the value of Equation (8) for 16QAM is larger, which indicates a worse NLI distortion, since the value of χ in Equation (13) for 16QAM is smaller compared to that for QPSK. Accordingly, based on the analytical model, the 16QAM system will outperform the 32QAM system, and the 32QAM system will outperform the 64QAM scheme. For DBP scenarios, the performance discrepancy of systems with different modulation formats is negligible due to the strong efficiency of DBP in the NLI mitigation. Comparing the NLC results in Figure 3a,b, it can also be found that the 32-GBd system performs better than the 64-GBd system, which indicates that the distortion from EEPN and ASE noises scales with the transmission symbol are rated as in Equations (1) and (3).

**Figure 4:**
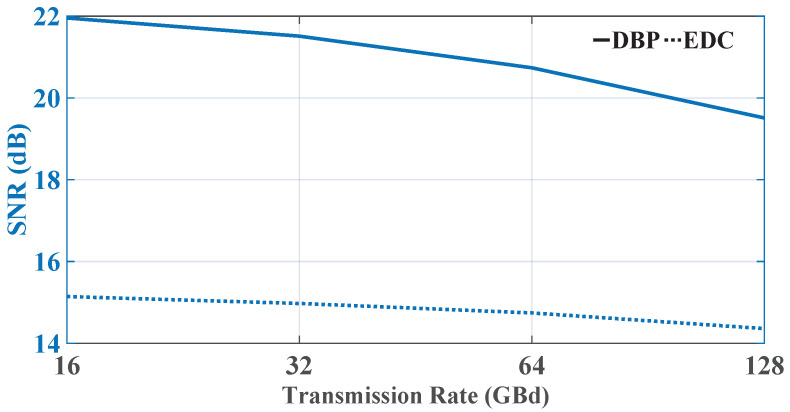
The central-channel SNR values in Nyquist-spaced WDM DP-16QAM nonlinear coherent fiber systems with transmission rates of 16, 32, 64 and 128 GBd, with transmission bandwidth fixed at 4.5 THz.

Next, the modulated bandwidth was extended to 4.5 THz to discuss the performance of an entire *C*-band system with different transmission symbol rate values. The performance of the 16-GBd 281-channel, 32-GBd 141-channel, 64-GBd 71-channel, and 128-GBd 35-channel DP-16QAM Nyquist-spaced systems at their optimum powers is shown in Figure 4. It is observed that, with the increase in the transmission symbol rate and the decrease in the channel number, the performance of the *C*-band Nyquist-spaced nonlinear fiber transmission with 100 kHz linewidth LPN gradually degrades when the overall transmission bandwidth is fixed at 4.5 THz.

**Figure 5:**
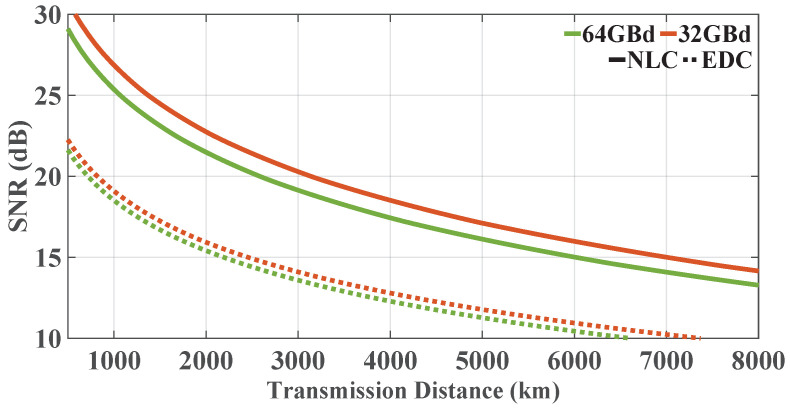
The central channel SNR with varying transmission distances in the Nyquist-spaced 5-channel DP-16QAM nonlinear coherent fiber system with symbol rates of 32 and 64 GBd at their optimum powers.

**Figure 6:**
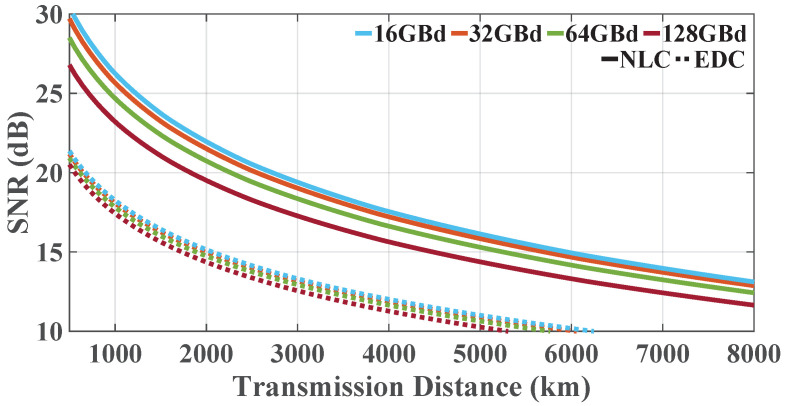
The central channel SNR with varying transmission distances in the wideband Nyquist-spaced DP-16QAM nonlinear coherent fiber system with transmission rates of 16, 32, 64, and 128 GBd at their optimum powers, where transmission bandwidth is fixed at 4.5 THz.

Figure 5 shows the center channel SNR taken at the optimum launch power values, calculated using the analytical model with varying transmission distances in the Nyquist-spaced 5-channel DP-16QAM nonlinear coherent fiber transmission, where transmission rates were 32 and 64 GBd. Figure 6 shows the analytical results in the systems with transmission rates of 16, 32, 64, and 128 GBd (281, 141, 71, and 35 channels, respectively) at their optimum powers, where the transmission bandwidth is fixed at 4.5 THz. From Figure 5 and Figure 6, it can be found that the system SNRs decrease with the increase in transmission distance, and systems with higher transmission rates suffer from heavier distortions. The SNR threshold of ∼15 dB for 16QAM (the BER threshold of $4.5\times 10^{-3}$ [29]) corresponds to the 7% overhead hard-decision forward-error-correction (FEC) error-free threshold [34], which can be employed as a benchmark. Figure 5 shows that, considering the 15 dB SNR threshold, the 32 GBd 5-channel system can transmit ∼1000 km longer than the 64 GBd 5-channel system in the case of NLC. The SNR values of the system employing EDC are higher than 15 dB when propagation distance is less than 2000 km. Figure 6 shows that, with the decrease in the channel number and the increase in the symbol rate, the C-band Nyquist-spaced system shows a worse performance. Figure 6 shows that, for *C*-band systems, considering an SNR threshold of 15 dB, the 16 GBd system can reach a ∼440 km longer transmission distance than the 128-GBd 35-channel system. The SNR values of the *C*-band systems employing EDC are more than 15 dB when distances are less than ∼1680 km.

## 6. Conclusions

The performance of wideband Nyquist-spaced long-haul multichannel nonlinear fiber systems influenced by the EEPN effect was analyzed and discussed based on both numerical simulations and analytical models. The efficiency and the accuracy of the presented model accounting for EEPN were demonstrated by simulations. The performance of *C*-band systems with different transmission symbol rates was also studied. The results indicate that the SNR of the *C*-band system using only EDC with 100 kHz linewidth LO LPN remains higher than 15 dB when the transmission distance is less than 1680 km, and that the 16 GBd system with NLC can reach a ∼440 km longer transmission distance than the 128 GBd system when considering an SNR threshold of 15 dB. This work provides insightful discussions for the design of high-speed wideband Nyquist-spaced WDM long-haul nonlinear fiber systems with considerable LPN.

## Figures and Tables

**Table 1:** System simulation setup parameters.

Parameters	Values
Attenuation coefficient	0.2 dB/km
Nonlinear coefficient (γ)	1.2 /W/km
CD coefficient (*D*)	17 ps/nm/km
Center wavelength	1550 nm
Channel spacing	{32,64} GHz
Symbol rate (*R*)	{32,64} GBd
Modulation format	{QPSK,16QAM}
EDFA noise figure	4.5 dB
Roll-off factor	0.1%
LO laser linewidth	100 kHz
Number of symbols	$2^{20}$

## Data Availability

Not applicable.
